# An Overview of Limonoids and Other Natural Products Isolated from the Medicinal Plant *Cipadessa baccifera* and Its Pharmacological Properties

**DOI:** 10.3390/plants15030466

**Published:** 2026-02-02

**Authors:** Christian Bailly

**Affiliations:** 1 UMR9020 CNRS, UMR1366 Inserm, CHU de Lille, Université de Lille, 59000 Lille, France; christian.bailly@univ-lille.fr; 2 Institute of Pharmaceutical Chemistry Albert Lespagnol (ICPAL), Faculty of Pharmacy, University of Lille, 59006 Lille, France; 3 OncoWitan, 59290 Lille, France

**Keywords:** *Cipadessa baccifera*, limonoids, medicinal plant, terpenoids

## Abstract

The Asian medicinal plant *Cipadessa baccifera* (Roth) Miq., also known as *C. fruticosa* or *C. cinerascens* (Ranabili or Nalbila), has long been used in Ayurvedic medicine to treat dysentery, skin disorders, rheumatism and parasitic infections. Extracts prepared from the leaves, seeds or fruits of the plant have revealed activities against mosquito vectors of parasitic diseases, notably marked larvicidal activities. Plant extracts have shown insecticidal and antibacterial activities, in addition to antioxidant effects. Numerous natural products at the origin of these pharmacological effects have been identified from all parts of the plant, from roots to leaves and seeds. The phytochemical survey presented here led to the identification of about 200 natural products isolated from *C. baccifera*, including a large majority of limonoids (>170), in addition to steroids, terpenoids, and a few other products. The panel of limonoids is extremely diversified with multiple groups of compounds: cipacinerasins, cipacinoids, cipacyclonone, cipadesins, cipadessains, cipadessalide, cipadonoids, cipafera, cipaferens, cipaferoids, ciparasins, cipatrijugins, cineracipadesins, cinerascenoids, and cipacinerasins. There are a few interesting bioactive products in *Cipadessa*, such as the anticancer agents cipaferen G and cipacyclonone, and the anti-inflammatory molecules cipadessain D and methyl-angolensate. Other bioactive products are discussed, such as cryptomeridiol, khayasin T, and febrifugin. An overview of *Cipadessa* phytochemicals is provided here to shed light on this under-valued medicinal plant.

## 1. Introduction

**Figure 1:**
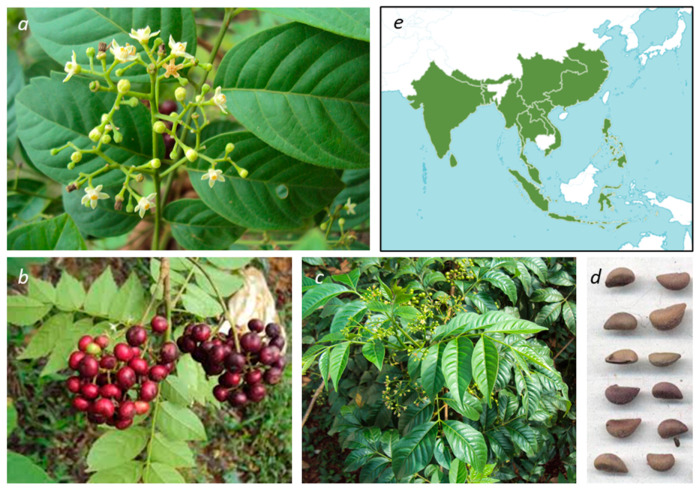
*Cipadessa baccifera* (Roxb. ex Roth) Miq. (**a**–**c**) aerial parts, flowers and fruits. From (https://commons.wikimedia.org/wiki/File:Cipadessa_baccifera_101.JPG), CC BY-SA 3.0. (**d**) seeds, reproduced with permission from Dr Kirill Tkachenko (V.L. Komarov Botanical Institute of the Russian Academy of Sciences, Saint-Petersburg, Russia) [6]. (**e**) geographic distribution of the plant (Source: https://www.worldfloraonline.org/taxon/wfo-0000605567, accessed on 27 January 2026) (CC BY 4.0).

Plants belonging to the *Cipadessa* genus are commonly used in folk medicine for the treatment of various diseases and conditions, including diabetes, rheumatism, and malaria, notably in India. *Cipadessa* leaf decoctions are also used to treat dysentery, headaches, and fevers. The paste of roots, leaves and bark is used topically to treat psoriasis and skin itches [1,2]. These plants are essentially distributed across India, southwest of China, Nepal and the Philippines [3]. *Cipadessa* plants have also been identified in west and central Malesia, Bangladesh, and the Bhutan Himalayas [4,5]. They can be found in most countries in Asia (Figure 1).

In fact, the genus is monotypic, with a single species, *Cipadessa baccifera* (Roxb. ex Roth) Miq., in the Meliaceae family. There are many synonym names for *C. baccifera*, such as *C. fruticosa*, *C. cinerascens*, *C. sinensis*, *C. subscandens*, and *C. warburgii* [7]. The species is endemic to the Western Ghats of India and Sri Lanka [8]. Different trial names are used for the plant, such as Ranabili (English name), Halbembiya (Sinhala name, in Sri Lanka), Pulippancheddi or Savattuchedi (Tamil name, in India), Pittamari (Odia, India), Nalbila (Hindi, India), and shael or dael (in the community of Bokod, Benguet, Philippines) [9,10,11]. The plant has been used in traditional medicine for a very long time. Early mentions of the use of *C. baccifera* appear in 16th-century Siddha manuscripts from Tamil Nadu (India), where it was called “Sippai maram”, used to treat intermittent fevers [12]. The plant is often combined with other medicinal species to treat snake bites, skin infections and other injuries [13].

*Cipadessa baccifera* is an evergreen woody shrub that propagates through seed and underground root stock. It is generally well distributed in open sun-lit areas along hill slopes and hill tops. Its hermaphroditic flowers are adapted for pollination by different classes of insects, such as bees, wasps and butterflies, which facilitate cross-pollination (xenogamy). The perennial plant produces fruits in the form of fleshy globular drupes, purple when mature, each containing 5–10 united ovoid seeds [6] (Figure 1). These fruits are consumed by frugivorous birds. Seeds can be dispersed by water (hydrochory), birds (ornithochory) or gravity near the parent plant (barochory), ensuring a prolific growth and a large distribution in open areas [14]. Beyond birds, the plant is also attractive to insects, notably for the Fagara silkworm (*Attacus atlas* Linn.) found in Karnataka, India [15]. The plant presents pinnately ellipsoidal leaves with extrafloral nectary glands, which secrete nectar until leaf maturity [16,17]. The plant can be identified through its morphological traits or using genetic markers. Its chloroplast genome has been fully sequenced [18]. Specific ISSR (Inter Simple Sequence Repeat) markers, based on DNA fingerprinting, have been characterized to facilitate the authentication of the plant [19].

The long-established traditional use of *C. baccifera* decoctions to treat various diseases has stimulated phytochemical research to identify natural products at the origin of the medicinal properties. Numerous bioactive products have been isolated and their properties characterized. The present review recapitulates research conducted over the past 40 years on this plant. The pharmacological properties of the plant extracts and their phytoconstituents are summarized. An overview of *C. baccifera* is provided.

The literature analysis was performed based on scientific articles retrieved from several databases (PubMed, Google Scholar, Scopus, Embase, Web of Science) and from sites of publishers (Elsevier, ACS, Biomed Central, NPG, and others). A comprehensive search was conducted across databases using keywords (*Cipadessa*, *C. baccifera*, natural products, limonoids) with no restrictions on publication date or language, although publications in the English language were mostly consulted.

## 2. Medicinal Uses of *C. baccifera* and Properties of Plant Extracts

### 2.1. Treatment of Parasitic Diseases

**Figure 2:**
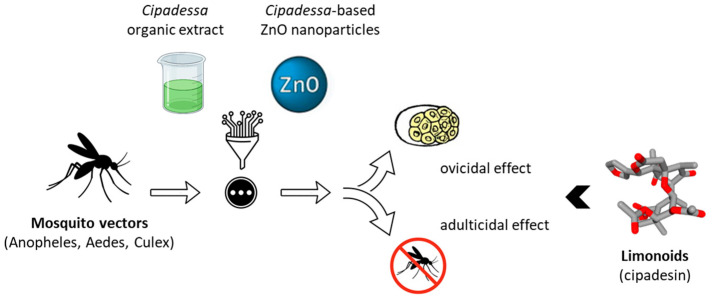
*Cipadessa* extracts have been used to inhibit mosquito vectors of parasitic diseases. Organic (notably acetone) extracts display marked activity against mosquitoes of the Anopheles, Aedes, and Culex genera, which are responsible for transmitting major vector-borne diseases. Stronger effects were observed with *C. baccifera* extracts prepared with zinc oxide-based nanoparticles (50–65 nm in size) [19,22].

Extracts of *C. baccifera* have not revealed direct activity against parasites, but they have shown beneficial effects to limit their dissemination through mosquito vectors. Organic extracts prepared from dried leaves of the plant were found to be active against three Diptera types of mosquito vectors: *Anopheles stephensi* (malaria vector), *Aedes aegypti* (dengue and Zika vector), and *Culex quinquefasciatus* (filariasis vector). The highest adulticidal activity was observed with an acetone extract and to a lower extent with methanol, ethyl acetate, chloroform and benzene extracts, with LD_50_ values in the range 23.5–29.6 mg/mL. A smoke toxicity assay using the leaf powder also revealed a potent activity against the three mosquito species [20]. Crude solvent extracts from *C. baccifera* display marked egg-hatching activities, in particular, acetone extracts, which showed the highest ovicidal activity [21]. In addition, silver- or zinc oxide-based nanoparticles prepared from *C. baccifera* extracts were found to exhibit a higher mortality rate compared to crude extracts. They can provide an eco-friendly approach toward the control of mosquito vectors at early stages [19,22,23] (Figure 2).

The larvicidal activity has been associated with the presence of limonoids, in particular, mexicanolide-type limonoids (see below) [24]. A petroleum ether extract of *C. baccifera* has been tested against *Culex* mosquito (*Culex quinquefasciatus*), which is the vector of lymphatic filariasis, but no major activity was observed [25]. A modest activity was noted with a methanolic leaf extract tested against two strains of *Plasmodium falciparum* [26]. Similarly, leishmanicidal activity was reported with methanolic extracts from the fruits, branches and leaves of *C. fruticosa*. The activity was evidenced using the nonpathogenic (reptile-associated) species *Leishmania tarentolae* and linked to inhibition of the enzyme adenine phosphoribosyltransferase (APRT). At that time, the bioactive fraction was not further analyzed to identify the natural products at the origin of the activity [27], but the activity can be associated with the presence of flavonoids and limonoids. A fruit extract of *C. baccifera* showed in vitro activity against the trypomastigote forms of *Trypanosoma cruzi* (responsible for Chagas’ disease), and the activity was associated with the presence of mexicanolide limonoids (febrifugin, cipadesin, cipadesin A) and flavonoids (7-methoxyflavone and 3′,4′,5′,5,7-pentamethoxyflavone) in the extract [28,29].

### 2.2. Antibacterial Activity

Organic extracts prepared from powdered leaves of *C. baccifera* have shown antimicrobial activities. The most potent effects were observed with a chloroform extract able to reduce the growth of *Pseudomonas aeruginosa*, *Shigella flexneri*, *Salmonella thyphi*, *Micrococcus* sp., and *Vibrio cholera*, at least to some extent [30]. Another study reported antibacterial activities with a methanol extract active against *P. aeruginosa*, but also *Klebsiella pneumonia* and *Enterococcus faecalis* [31]. The antibacterial action was reevaluated recently, and a potent activity was observed with an ethyl acetate fraction of a leaf extract, essentially active against the Gram-positive bacteria *Staphylococcus aureus* and the Gram-negative *Salmonella typhi* [32]. The natural products at the origin of this antibacterial activity have not been identified, but two independent studies evidenced the presence of antimicrobial triterpenoids and steroids in the branches and leaves [33] and antimicrobial limonoids in the seeds of the plant [33]. However, the level of antimicrobial activity proved to be very modest compared to what can be achieved with aminoside antibiotics. Cinerascenoids A-C were found to be 20- to 100-fold less potent against pathogenic microorganisms than gentamycin, for example [34].

Interestingly, a broad-spectrum antibacterial activity was reported with a root extract of *C. baccifera*, notably with a methanol extract active against bacteria responsible for diarrhea (*E. coli*, *S. flexneri*, and, to a lower extent, *B. cereus*), skin and wound infections (*Pseudomonas aeruginosa*, *Pseudomonas cepacia*, and *Propionibacterium acnes*), and oral infections (*Streptococcus gordonii* and *S. mutans*, *Corynebacterium diphtheriae*). In the same study, an essential oil of *C. baccifera* roots exhibited moderate antimicrobial activity but marked action against *E. coli* [35]. The essential oil obtained from the plant leaves essentially contained sesquiterpenes, notably caryophyllene, isoledene, *cis*-calamenene and β-sesquiphellandrene [36].

### 2.3. Other Activities

Two studies have underlined the capacity of *C. baccifera* extracts to reduce the growth of cancer cells in vitro. The first study only cited the cytotoxicity of the plant extract against three cancer cell lines (HeLa, Jurkat, MCF-7, and KB cells) [37]. The second one compared the cytotoxic activity of methanolic extracts prepared from the roots, stem bark, leaves and fruit of the plant. The most potent effects were observed with the leaf and root extracts on MCF-7 and KB cell lines [38].

Unsurprisingly, antioxidant effects have been observed with the total extract and methanolic fractions of the plant, also able to inhibit xanthine oxidase [39,40]. A marked activity was reported when using an ethanolic defatted extract (EDE), potently acting as a scavenger for oxygen-based free radicals. This EDE fraction was tested in vivo in a model of wound healing in diabetic rats. At the oral doses of 100 and 200 mg/kg, the product showed 75 and 95% wound closure, respectively. The activity was associated with the presence of alkaloids, terpenoids, flavonoids, saponins and tannins [41,42]. The activity was not spectacular but significant and certainly useful, considering that it is a safe product. No acute oral toxicity was observed in rats up to 2000 mg/kg body weight [43]. A few other bioactivities have been evidenced, notably a modest thrombolytic activity with organic extracts from the plant leaves [44].

Insecticidal activity has been reported with an organic extract of *C. baccifera*, notably when methanol was used as the extraction solvent. The MeOH extract showed a modest activity against the invasive pest fall armyworm (*Spodoptera frugiperda* (J. E. Smith)), which causes major losses in global crop production. This highly invasive lepidopteran pest poses a significant threat to global maize production worldwide. However, the level of activity was very modest, inferior to that observed with another Meliaceae plant, *Cedrela fissilis* Vell. [45]. Similarly, a weak activity has been observed with a crude organic extract against the Lepidopteran *Helicoverpa armigera* (Hubner), which is also a global pest of many major crops. A 0.5% acetone extract showed an ovicidal effect, blocking hatchability of 24 h old eggs [46]. The fecundity and egg hatchability were reduced, notably when the *C. baccifera* extract was combined with another plant extract (from *Argemone mexicana*) active against the American bollworm (*Helicoverpa armigera*) [47]. A non-medicinal but ecological use of *C. baccifera* has been reported. It consisted of the use of the plant to facilitate the degradation of the commonly used pesticide endosulphan (a persistent organochlorine pesticide). Alone or mixed with cow dung, the plant promoted the chemical transformation of the pesticide over a period of 30–90 days [48]. A few other bioactivities have been reported with *Cipadessa* extracts, such as the treatment of snake bites and infections by pathogenic yeasts (candidiasis) [13].

Collectively, the data underline the utility of *Cipadessa* extracts to combat certain parasitic diseases, bacterial infections, or pathologies associated with oxidative damage. These effects have lent credence to the traditional uses of the plant to treat malaria or to alleviate rheumatism. The natural products at the origin of these therapeutic effects are discussed below.

## 3. Natural Products Isolated from *C. baccifera* and Syntheses

A previous survey (in 2012) of the natural products isolated from four *Cipadessa* species (*C. baccifera*, *C. fruticosa*, *C. cinerascens*, *C. boiviniana*) referred to 101 secondary metabolites, with a large majority of terpenoids (including 56 limonoids, 15 sesquiterpenoids, three diterpenoids), in addition to different steroids (15), flavonoids (6), and a few other products [49]. In fact, *C. baccifera*, *C. fruticosa*, and *C. cinerascens* refer to the same plant, now designated *Cipadessa baccifera* (Roxb. ex Roth) Miq., which is the accepted name; the other names are considered synonyms [7]. It is distinct from *Cipadessa boiviniana*, which is a synonym for the plant *Malleastrum boivinianum* (Baill.) J.-F.Leroy, found in Madagascar. Here, the analysis is limited to the products isolated from *Cipadessa*, i.e., *C. baccifera* and the two synonyms.

An in-depth analysis of the scientific literature pertaining to this plant led to the identification of 220 natural products cited in 39 publications over the period 2000–2025. All parts of the plant have been used to search for new natural products, from roots to aerial parts. The leaves and seeds of *C. baccifera* have been primarily exploited to search for new natural products (Table 1). Both the leaves and roots of the plant have been used in Chinese folk medicine for the treatment of different pathologies, but thus far, phytochemical studies have been essentially performed using the aerial parts of the plant, rarely the roots.

It is particularly tricky to navigate through the multiple groups of products isolated from this plant, notably because of the redundancy in the names given to those series of products. The reader can be rapidly confused between the numerous products with homonymous names, such as the cipacinerasins, cipacinoids, cipacyclonone, cipadesins, cipadessains, cipadessalide, cipadonoids, cipafera, cipaferens, cipaferoids, ciparasins, and cipatrijugins, in addition to the cineracipadesins, cinerascenoids, and cipacinerasins. Most of them are structurally related limonoids, and confusion between the products can be easy in the case of homonymy. The naming of new natural products is a key exercise, with specific rules [50]. In the “cipa-” family, the situation is particularly complex, with many closely related limonoids. It is well known that the structural diversity is very large among limonoids. In 2021, Luo and coworkers reported about 2700 meliaceous limonoids, including a small number (about 30) from *Cipadessa* [51]. In 2022, Mulani and coworkers identified 1502 Meliaceae limonoids classified into 57 groups, with specific mention of about 100 limonoids from *Cipadessa* species [52]. Limonoids play an important role in health and also in the agriculture and food industries. They can be extracted from many plants, particularly from Meliaceae and Rutaceae [53,54,55].

**Figure 3:**
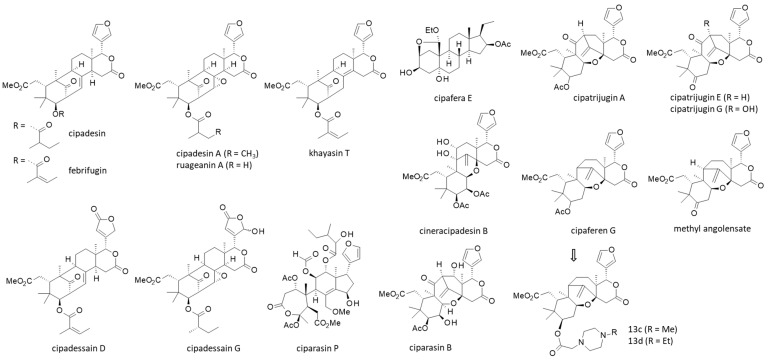
Chemical structures of selected *Cipadessa* limonoids. The family includes about 170 natural products, with multiple series as indicated (see Table 1 for more details about the products and their organ origin).

In the present case, a careful analysis of the phytochemical data pertaining to *Cipadessa* led to the identification of 171 limonoids, 15 terpenoids, 27 steroids, and a few other products (alkanes, flavonoids). Strangely, the presence of alkaloids and saponins in *Cipadessa* extracts has been mentioned in different publications [10,31,32,56], but there is no study describing specific alkaloids isolated from this plant. *Cipadessa* contains a large diversity of limonoids, which are highly oxygenated tetracyclic triterpenoids, and smaller compounds with a tri- or bicyclic core, such as the sesquiterpene voleneol and a hydroperoxyl derivative [57]. Among *Cipadessa* limonoids, the structural diversity is large, notably with trijugin-, mexicanolide-, havanensin-, phragmalin-, prieurianin- and cipadesin-type limonoids [58]. Chemical structures for representative members of *Cipadessa* limonoids are shown in Figure 3. Intact and rearranged limonoids coexist in the plant. Some are relatively common, such as gedunin and febrifugin, which are often found in Meliaceous plants [59]. Others are much more specific, if not exclusive to *Cipadessa*, such as cipadessalide or the cyclopentenone derivative cipacyclonone, only found in this plant [59].

**Table 1:** Natural products isolated from *C. baccifera*.

Plant Parts	Products	Ref.
Seeds	Cipadesin, 17α,20R-dihydroxypregnan-3,16-dione, 1,4-epoxy-16-hydroxyheneicos-1,3,12,14,18-pentaene, 1,4-epoxy-16-hydroxyheneicos-1,3,12,14-tetraene, 2β,3β,4β-trihydroxypregnan-16-one, febrifugin, khaysin T	[60]
	Tigloylseneganolide A, 2′R-/2′S-methylbutanoylproceranolide, 2′R-/2′S-cipadesin A, 2′R-/2′S-cipadesin, ruageanin A, swietemahonolide, febrifugin, methyl 3α-isobutyryloxy-1-oxomeliac-8,30-enate, khayasin T, and 3α-isobutyryloxymexicanolide	[61]
	Cipaferen E,F,G,H,J,K,L,M. Granatumin E, 2′R-methylbutanoylproceranolide, khaysin T, 2′R-cipadessin A, febrifugin, xylomexicanin B	[62]
	17α,18,20S-trihydroxy-pregn-4-en-3,16-dione, 18-hydoxy-pregn-4,17(20)-trans-dien-3,16-dione, 3β,18-dihydroxy-pregn-5,17(20)-trans-dien-16-one, 2α,3β,4β,18-tetrahydroxy-pregn-5,17(20)-trans-dien-16-one, 2α,3β,4β,17α,18,20S-hexahydroxy-pregn-5-en-16-one, 17α,20S-dihydroxy-pregn-4-en-3,16-dione, 3β-hydroxy-pregn-5,17-dien-16-one	[63]
	Cipaferen E,G,H,K,L. Khaysin T, methylbutanoylproceranolide	[64]
	Cinerascenoids A-C	[34]
	Cipadesin, febrifugin	[65]
	Cipaferen G,N,O. 3-*O*-deacetylcipaferen G	[66]
Aerial parts	Cipadesins A-C	[67]
	Cipatrijugins A,G	[68]
Branches	Cineracipadesin G, cineracipadesin C, cineracipadesin D, cipatrijugin B, cipatrijugin A, cipadonoid C, 3-deacetylcipadonoid D, cipadonoid D, (+)1β,6α-dihydroxy-4(15)-eudesmene, cineracipadesin G, 1β-hydroperoxy-6α-hydroxy-eudesm-4(15)-ene	[57]
Branches and leaves	Cipacinerasins A-K. Cipadonoid A, cipadesins C-F,Q, ciparasin A, cipatrijugins E-F,H, ciparasins D,M, methyl angolensate	[69]
	Cipafera A-E. Cipadesin B, ciparasin H, 21α-methylmelianodiol, 21β-methylmelianodiol, melianodiol, 2*α*,3*β*-dihydroxy-16,17-seco-pregn-17-ene-16-oic acid methyl ester 2β, 2α,3β-dihydro-5-pregnen-16-one, 2*β*,3*β*,4*β*-trihydroxypregnan-16-one, 1-methoxy-pregnan-17(*R*)-1,4-dien-3,16-dione, 1-methoxy-pregnan-17(*S*)-,4-dien-3,16-dione	[33]
Stems	Cipadessalide, rubralin D, 3β,4β-dihydroxy-2β-acetoxypregnan-16-one, and bacciferins A-B. Mombasol, 2β,3β,4β-trihydroxypregnan-16-one, 2β,3β-dihydroxy-5α-pregnan-16-one, 3β,16α,18*S*-trihydroxypregnane, meliavosin, 9β-hydroxyaphanamol II, guaianediol, cryptomeridiol, 4(15)-eudesmene-1β,6α-diol	[70]
	Cipadesin D,E	[71]
	Lanost-7-en-3-one-22,25-epoxy-23,24-acetone acetal, chisopanin M, 3β-hydroxy-5-en-stigmast, 7α-hydroxy-4-en-3-one-stigmast, 3β-hydroxy-5-en-7-one-stigmast, 7α-hydroxysitosterol, 22*E*-7α-methoxy-5α,6α-epoxy-8(14),22-dien-3β-hydroxy-ergosta, 7β-hydroxy-4-en-3-one-cholest, 3β-acetyloxy-2β,4β-dihydroxy-16-one-pregnan, 17α,20R-dihydroxypregnan-3,16-dione, 2β,3β-dihydroxy-16-one-pregnan, 3β,7α-dihydroxy-20-one-pregnan, 2α,3β-dihydroxy-5-en-20-one-pregnan, 1,4-dien-3,16-dione-2-hydroxyandrosta, 5-en-17-one-3β,16β-dihydroxyandrost, apigenin, annuionone D	[72]
Stem bark	Cipatrijugins A,D,E,F	[73]
Leaves and bark	Cipadesins D-F, 8,15-dihydroxy-13E-labdane, β-sitosterol, β-daucosterol	[74]
	Cipadesin A-F	[75]
Leaves	Cipatrijugins A-D, cipadesin A	[76]
	Cipadonoid A-G	[77,78]
	Cineracipadesins A-F, methyl 2β,3β-diacetoxy-3-deoxoangolensate	[79]
	Gedunin, havanensin, mexicanolide, methyl angolensate, cipadesin J-Q	[73]
	Ciparasins A-P	[80]
	Cipacyclonone	[59]
	Cipacinoids A-D	[81]
	Cipacinoids E-O	[82]
	Cibacciferin A-I, 11α-acetoxycibacciferin A, 2′-epi-cibacciferin B-C, 11α-acetoxycibacciferin C, 2β-acetoxycibacciferin E, 6-dehydroxycibacciferin F, 12-deacetoxycibacciferin E, 2β-acetoxy-12-deacetoxycibacciferin E, 12-dehydroxycibacciferin H	[83]
	Cpd 1 and cipadesin-type limonoids 2-3	[84]
	Cipatrijugins G, H, cipadesin F, cineracipadesin A	[85]
	Cipaferens A-D, melianodiol, spicatin	[86]
	Cipadesins G-I, cipadesin B,C,D, cineracipadesin E	[87]
Twigs and leaves	21-deoxo-23-oxofebrifugin A, 3-O-detigloyl-3-O-isobutyryl-21-deoxo-23-oxofebrifugin A, 3-O-detigloyl-3-O-isobutyrylfebrifugin A, febrifugin A, febrifugin, khaysin T, Cipaferen R, granatumin E, khaysin T, 2′S-cipadesin A	[88]
	Cipaferoids A-C	[89]
Fruits	3-de(2-methylbutanoyl)-3-propanoylcipadesin, febrifugin, khayasin T, 20S-cipadesin, methyl-8a,30a-epoxide-3b-(20-methylbutyryloxy)-1-oxomeliacate, xylocarpin, swietemahonolide, swietemahonin F, cipadesin D, cipadesin H, cipadesin I, mesendanin T	[90]
	Cipadesin, cipadesins A-B, febrifugin, febrifugin A, khayasin T, ruageanin A, mexicanolide	[91,92]
	Cipadessains A-K	[93]
	Cipadesin A, ruageanin A, cipadesin, febrifugin, febrifugin A, khayasin T	[94]

**Figure 4:**
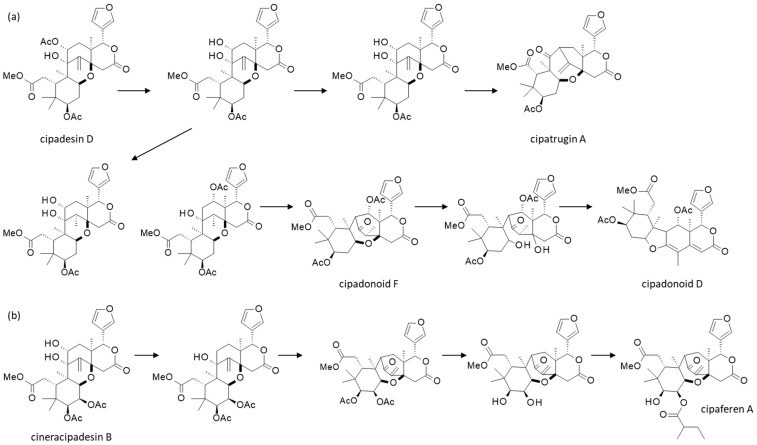
The hypothetical biogenetic pathways proposed (**a**) from cipadesin D to citrijugin A, or cipadonoids D and F [97] and (**b**) from cineracipadesin B to cipaferen A [62].

Many of these *Cipadessa* limonoids are derived from the same biosynthetic pathway. For example, a biogenetic route from cipadesin D to cipatrijugin A and cipadonoid D/F has been proposed (Figure 4) [77]. Similarly, cipaferen A may derive from cineracipadesin after a short series of transformations (pinacol rearrangement, hydrolysis, and esterification) [62]. The biosynthesis of limonoids has been partially elucidated; many steps remain unknown. It must be said that the biosynthetic routes are numerous. It has been estimated that a network of 20,000 triterpenoid structures can be generated from the precursors farnesyl diphosphate and squalene [95,96]. Twenty-two limonoid biosynthetic genes have been identified, encoding 12 distinct chemical transformations, such as scaffold rearrangements, acetylations and oxidations [97].

**Figure 5:**
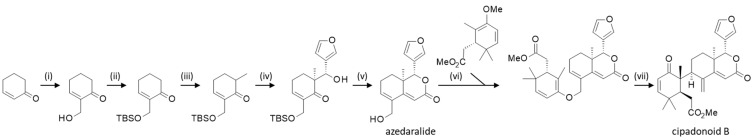
Synthesis of cipadonoid B from 2-cyclohexenone, via the intermediate azedaralide [102,104]. Conditions: (i) HCHO, base; (ii) TBDMSCl; (iii) LDA and then MeI; (iv) potassium bis(trimethylsilyl)amide (KHMDS) in THF at −78 °C, then (−)-diisopinocampheylchloroborane [(−)-DIP-Cl], and then 3-furylaldehyde; (v) Ac_2_O, DMAP, pyridine, and then lithium diisopropylamide (LDA) in THF at −78 °C, then rt; tetrabutylammonium fluoride (TBAF) in THF at −20 °C, to obtain azedaralide; (vi) reaction of azedaralide with the vinyl ether derivative ((S)-methyl 2-(3-methoxy-2,6,6-trimethylcyclohexa-2,4-dien-1-yl)-acetate afforded the enol ether intermediate, precursor to enantiopure (-)-cipadonoid B via a ketal−Claisen cascade (vii) (see [100] for complete details).

Some of these products can be accessed by chemical synthesis, as reported for the tetracyclic limonoids cipadonoids A and B [98,99]. A gram-scale stereoselective synthesis of cipadonoid A, starting from 2-cyclohexenone in 16 steps, with an overall yield of about 3%, has been proposed recently [100,101]. Similarly, the synthesis of cipadonoid B from the synthetic precursor azedaralide (itself prepared from (+)-α-pinene or cyclohexanone) has been described [99] (Figure 5). But in general, limonoids are not easily accessible by chemical synthesis due to their complex asymmetric structures. Despite recent progress, the de novo construction of limonoids remains a challenging problem [102,103,104]. A few compounds, such as gedunin, can be accessed by chemoenzymatic synthesis [105], but most compounds need to be isolated from nature for pharmacological studies.

## 4. Pharmacological Properties of Cipadessa Natural Products

One of the first limonoids isolated from *Cipadessa* twenty-six years ago was cipadesin. This tetracyclic compound, structurally close to febrifugin and khaysin T, also found in the plant, is emblematic of the species (Figure 3). It is the leader product of the so-called cipadesin-type limonoids, which include cipadesin A-Q, ciparasins H-O, and other related products [75,80]. Cipadesin differs from cipadesin A, which bears an epoxide group. Interestingly, this latter product has revealed anti-depressant activity in mice, linked to inhibition of the hypothalamus–pituitary–adrenal axis activity response to stress. This type of effect is of interest in treating depression and anxiety disorders [106]. It would be useful to evaluate the activity of ruageanin A, which is very close to cipadesin A, and to compare the effect of the 2′R and 2′S epimers, both present in the plant [61].

Cipadesin, febrifugin and related products also present moderate antiparasitic activities (IC_50_ > 100 µM), notably against the trypomastigote forms of the parasite *Trypanosoma cruzi* responsible for Chagas disease [29]. Limited anti-trypanosomal activity has also been reported with three flavonoids (flavone, 7-methoxyflavone and 3′,4′,5′,5,7-pentamethoxyflavone) isolated from *Cipadessa*. The targeting of the *T. cruzi* glycosomal glyceraldehyde-3-phosphate dehydrogenase (gGAPDH) enzyme has been suggested, but the level of activity is excessively limited (IC_50_ > 200 µM) [28]. Cipadesins have also been tested against the malaria parasite using the chloroquine-resistant Dd2 strain of *Plasmodium falciparum*, but only a modest activity was observed, in the 16–28 µM range, compared to an nM activity with the reference artemisinin [83]. Overall, the antiparasitic activity of *Cipadessa* limonoids is limited.

Many limonoids isolated from *Cipadessa* have been characterized structurally but not pharmacologically. This is the case for the cipadesins and cipadessalide; notably, their pharmacology is essentially unknown [70,71,74,76]. Others have been evaluated as cytotoxic agents, such as the cipacinerasins, but the level of cytotoxicity was found to be very modest [79]. *Cipadessa* limonoids are not potent cytotoxic products in general. Only a moderate cytotoxicity toward leukemia (K562, HEL), prostate (LN-Cap, PC3), and cervical (HeLa) cancer cells was noted with cipafera E (IC_50_ in the range 6–20 µM) [33]. Cipatrijugin G proved to be cytotoxic to A549 lung adenocarcinoma cells (IC_50_ = 9.78 μM) [68]. In the same vein, cipatrijugin E and cipadesin K revealed a modest cytotoxic activity against human liver (SMMC-7721) cancer cells (IC_50_ = 21.6 and 36.5 µM, respectively) [73]. The cipaferens also showed modest antiproliferative properties [62,66,89]. But the anticancer mechanism of action is not known. In a few cases, potential targets have been proposed, such as the protein tyrosine phosphatase 1B (PTP1B), which can be targeted with cipacinoid A (IC_50_ = 16.7 μM), but the level of activity is modest compared to what can be achieved with known (nM-active) PTP1B inhibitors [81].

The limonoid cipaferen G is an interesting compound because it showed a marked cell-type-dependent cytotoxicity. It was found to be inactive against MDA-MB-231 breast cancer cells, moderately active against PANC-1 pancreatic cancer cells, and highly potent against IMR-32 neuroblastoma cells (IC_50_ = >100, 16.7, and 0.022 μM, respectively). Synthetic derivatives of cipaferen G have been prepared, notably compounds incorporating a methyl- or ethyl-piperazine unit with increased solubility and activity (cpds 13c-d in Figure 3, IC_50_ = 0.04 and 0.013 μM, with IMR-32 cells) [66]. The esterification of cipaferen G represents a convenient option for the design of new (and patentable) compounds with reinforced drug-like properties. Aliphatic and aromatic groups can be introduced on the limonoid core scaffold without losing activity.

**Figure 6:**
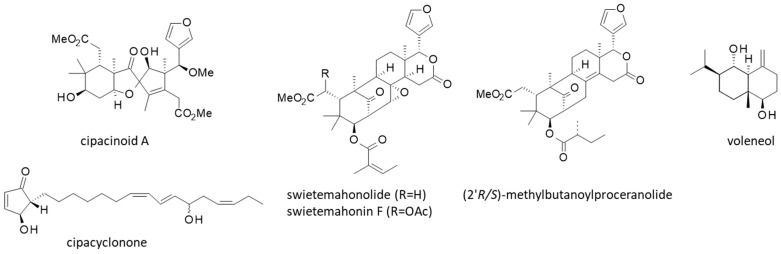
Other natural products isolated from *Cipadessa*.

Cytotoxic activities have also been observed with pregnane-type steroids isolated from *Cipadessa*, but the level of activity was relatively modest. The best compound in the series (2α,3β,4β,17α,18,20S-hexahydroxy-pregn-5-en-16-one) gave IC_50_ values in the range 7.1–9.5 µM depending on the cancer cell line, compared to 15.1–30.1 nM with the reference product doxorubicin [63]. By comparison, the cyclopentenone derivative cipacyclonone (Figure 6) proved to be significantly more active, with cytotoxicity values in the low micromolar range (IC_50_ = 1.2 and 3.0 µM with HL-60 leukemia cells and A549 lung cancer cells, respectively) [59]. This rare compound deserves further studies to understand its mechanism of action.

**Figure 7:**
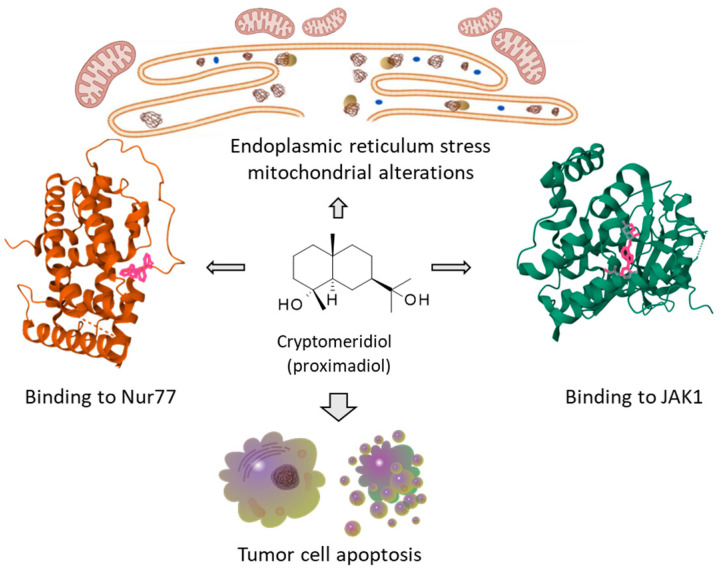
Cryptomeridiol and its effects on cancer cells. The binding of this sesquiterpene to nuclear receptor Nur77 and/or Jak1 kinase triggers endoplasmic reticulum stress and mitochondrial dysfunctions, leading to apoptosis, notably in hepatocellular carcinoma cells [108,109,110,111].

The interest in *Cipadessa* is not limited to its limonoid content. A few other active anticancer compounds have been identified from the plant, such as the sesquiterpene diol cryptomeridiol isolated from stem extracts [70]. This small molecule, also known as proximadiol [107], has been shown to target the orphan nuclear receptor Nur77 and to induce endoplasmic reticulum (ER) stress, mitochondrial dysfunction and apoptosis of hepatocellular carcinoma (HCC) cells. Remarkably, this bicyclic sesquiterpenoid showed a potent dose-dependent antitumor effect in mice bearing HepG2 xenograft tumors [108]. Cryptomeridiol is active against HCC and other tumor cell types, notably gastric cancer cells [109,110]. The compound can bind to Nur77 and has been predicted to interfere with the JAK-STAT signaling pathway, possibly via a direct interaction with kinase Jak1 [111] (Figure 7). Cryptomeridiol is also an antibacterial agent [112] and a potential binder to *Plasmodium falciparum* lactate dehydrogenase (*Pf*LDH) [113]. This eudesmane-type sesquiterpene diol, biosynthesized from farnesyl diphosphate (FPP) via hedycaryol [114], can be found in diverse medicinal plants, including in the form of glycosides (e.g., sonneratioside A) [115]. The synthesis and biotransformation of new cryptomeridiol derivatives warrant further investigations [116].

In the cipadessain series, the two compounds cipadessains C and F showed potent anti-inflammatory action but proved to be cytotoxic. In contrast, cipadessains D and G were a little less potent at inhibiting the production of nitric oxide in murine LPS-activated RAW 264.7 macrophages but presented no significant cytotoxicity (NO production inhibition: IC_50_ = 5.79, 23.90, 6.93, and 20.54 μM, for cipadessains C, D, F, and G, respectively) [94]. The limonoid methyl-angolensate (MA) found in *Cipadessa* seeds and leaves, and in many other plants, also displays potent anti-inflammatory action [62,117,118]. This tetranortriterpenoid is a pro-apoptotic agent, functioning as an activator of DNA damage in cancer cells by inducing the activation of DNA double-strand break repair proteins, such as the Ku70/Ku80 heterodimer complex, which plays a central role in the non-homologous end joining (NHEJ) double-strand break (DSB) repair pathway and the DNA damage response (DDR) [119]. Analogous limonoids, such as cineracipadesin B with a methyl angolensate-type structure [79], may function in a similar manner. MA is an interesting compound, all the more so because it also displays sedative properties, with a capacity to prolong the duration of pentobarbital-induced sleeping time in rats [120], coupled with an inhibitory effect on gastrointestinal smooth muscle contraction [121]. For a long time, MA has been considered as a natural product of interest to prevent gastric ulceration and to treat peptic ulcers [122,123].

Finally, antiviral effects have been reported with different *Cipadessa* limonoids, notably with ciparasins B and P, which revealed anti-HIV activities (EC_50_ = 5.5 and 6.1 μM, respectively) [82]. They are not extremely potent, but they can provide a useful starting basis for chemical modifications [103]. A few products with insecticidal and/or antifeedant activities have been identified in the plant. This is the case for methylbutanoylproceranolide (Figure 6) isolated from the seeds of the plant [61,62,64]. The 2′S-isomer of the compound has shown marked insecticidal activity against fifth-instar larvae of the coconut palm pest *Brontispa longissimi* (Coleoptera) [124]. Insecticidal effects have also been reported with *Cipadessa* limonoids, notably with cipadesin A, khayasin T, febrifugin, and febrifugin A [24,94]. The two tetranortriterpenoids swietemahonin F and swietemahonolide, which have been isolated from *Cipadessa* fruits [91,92,93], likely contribute to the insecticidal action. Swietemahonin F has shown antifeedant action against *Spodoptera frugiperda* in an antifeedant assay using final instar larvae [125].

## 5. Discussion

The monotypic plant species *Cipadessa baccifera* (synonyms: *C. fruticosa*, *C. cinerascens*) has been used for a long time to treat human diseases, notably in India. It is a classical Ayurvedic plant used to alleviate digestive discomfort and inflammation. Health-beneficial effects have been reported using different parts of the plants, mostly the leaves and fruits, used for mild digestive preparations. Extracts of *Cipadessa* have revealed good but relatively modest antibacterial effects and anti-inflammatory properties. The insecticidal effects observed with organic (acetone) leaf extracts are of interest to limit the dissemination of parasitic diseases by mosquito vectors. Encouraging data have been reported with *Cipadessa*-based nanoparticle preparations to limit disease transmission [22]. There is now a large range of data that support the use of Meliaceae plant extracts as biopesticides. There is an active development of eco-friendly formulations co-assembled with limonoid-based nanoparticles [126]. This type of limonoid-rich extract can be extremely useful in inducing potent and durable mosquitocidal effects; they function as ovicides, larvicides, and/or oviposition repellents [127]. Encouraging data have been reported not only with *Cipadessa* but also with other Meliaceous plants [128]. In this context, the use of *Cipadessa* extracts and preparations containing purified limonoid should be encouraged.

*Cipadessa* presents a very rich limonoid profile, with more than 170 distinct limonoid compounds identified thus far. They are polycyclic oxygenated products, structurally complex but well characterized. The structural complexity is usually not a major obstacle to the structural determination of these natural products. There are robust analytical techniques and methods (e.g., 1D/2D-NMR, HR-MS, LC-MS-MS, X-ray crystallography) to help establish structures of new organic molecules [129,130,131,132]. The structural diversity among *Cipadessa* limonoids is large, with compounds comprising a cipadesin-type (e.g., ciparasins H-O), mexicanolide-type (e.g., cipaferen K-M) or a trijugin-type (e.g., ciparasins A-G) structure or a spirocyclic skeleton (e.g., cipacinoids A-D) [81,133]. Some of these compounds can be accessed by total synthesis, such as cipadonoids A-B [100], but in most cases, the natural products need to be extracted from the plant. The chemistry of limonoids is relatively well developed, with more than 800 limonoid derivatives synthesized thus far [103] and new scaffolds elaborated [134]. But efforts must continue to facilitate access to complex natural products. Similarly, the biosynthesis of *Cipadessa* limonoids is only partially understood. Biosynthetic pathways leading to cipadonoids and cipaferen have been proposed [79,87]. Several processes, enzymes, and genes implicated in the construction of protolimonoid precursors and furan moiety of limonoids have been identified recently [135,136,137,138]. More work is needed to dissect the construction process for these complex molecules more precisely.

At the pharmacological levels, data are very limited, but a few encouraging results have been reported to better comprehend the mechanism of action of some *Cipadessa* limonoids. The capacity of methyl-angolensate to activate DNA double-strand break repair proteins so as to regulate DNA damage in cancer cells provides important elements to exploit the compound in cancer research [119]. Other limonoids, such as nimbolide, have been shown to regulate the DNA damage response in tumor cells [139]. These compounds may function as PARP1 (poly ADP-ribose polymerase 1) inhibitors or modulators [140]. Nomilin can form stable complexes with PARP1 and also with other proteins implicated in tumor development, such as Bcl2 and Hsp90 [141]. Nimbolide, like other limonoids, elicits pleiotropic effects on cancer cells, acting on several signaling pathways [142]. A better understanding of the potential targets for *Cipadessa* limonoids is absolutely needed to exploit the bioactive compounds. In the field of cancer, the interest in the plant is not limited to its limonoids. There are a few other compounds of interest, such as the sesquiterpene cryptomeridiol targeting the nuclear receptor Nur77 in hepatocellular carcinoma [108]. In other words, the pharmacological interest in *Cipadessa* cannot be associated with one series of compounds in particular, but with the diversity of products present in the plant and its extracts. The numerous limonoids, diverse terpenoids, steroids, and flavonoids present in *Cipadessa* all contribute to the plant’s attractivity [13]. The plant is of interest in medicine but also in dermo-cosmetics. An under-eye herbal cream formulated with a *Cipadessa* fruit extract is commercially sold as a beauty product to reduce eye wrinkles. This activity may be associated with the antioxidant and anti-aging properties reported with *Cipadessa* extracts [39,40]. Patents covering cosmetic compositions that include *Cipadessa* extracts are also emerging (e.g., US20250127745A1). The plant is gaining interest and with no doubt “Limonoids are on the menu” [97], but more work is needed to further characterize the key active molecules pharmacologically. Notably, more in vivo studies are needed to confirm the efficacy and safety of the best active compounds in vitro. At present, animal studies are limited, and no clinical testing of *Cipadessa*-derived products has been performed. Limoinoids (e.g., nimbolide) often present poor solubility and limited bioavailability, constraining their in vivo uses. Studies on the bioavailability and pharmacokinetic characteristics of limonoids, as done with limonoids like nimbolide and obacunone, for example [143,144], should be encouraged.

## 6. Conclusions

The large chemical diversity and the presence of diverse bioactive natural products, mainly limonoids, provide support to the long-established ethnobotanical use of Ranabili (*Cipadessa baccifera*) in traditional Indian medicine. This medicinal plant, belonging to the Meliaceae (Mahogany) family, is a true limonoid reservoir. Structurally diverse trijugin-, mexicanolide-, havanensin-, phragmalin-, prieurianin- and cipadesin-type limonoids have been isolated from the aerial parts of the plants and the fruits and seeds. The plant also contains terpenoids and steroids of interest, but the pharmacological properties of all these compounds have been under-investigated thus far, perhaps due to limited (bio)chemical access to these compounds. A few compounds of prime interest are emerging, such as cipaferen G, methyl-angolensate and cryptomeridiol. But more work is needed to better appreciate the pharmacological and therapeutic potential of this medicinal plant.

## Data Availability

No new data were created.
